# Seabird Modulations of Isotopic Nitrogen on Islands

**DOI:** 10.1371/journal.pone.0039125

**Published:** 2012-06-18

**Authors:** Stéphane Caut, Elena Angulo, Benoit Pisanu, Lise Ruffino, Lucie Faulquier, Olivier Lorvelec, Jean-Louis Chapuis, Michel Pascal, Eric Vidal, Franck Courchamp

**Affiliations:** 1 Estación Biológica de Doñana. Consejo Superior de Investigationes Científicas (CSIC), Avda. Americo Vespucio, Sevilla, Spain; 2 Muséum National d’Histoire Naturelle, Département EGB, Paris, France; 3 Department of Biology, Section of Ecology, University of Turku, Turku, Finland; 4 Société d’Ornithologie de Polynésie, Tahiti, Polynésie Française; 5 INRA, INRA and Agrocampus Ouest: Écologie et Santé des Écosystèmes, Campus de Beaulieu, Bâtiment, France; 6 IMBE, Aix-Marseille University, Centre IRD de Nouméa, Nouméa, New-Caledonia; 7 Laboratoire ESE, University Paris, Paris, France; Toulouse, France

## Abstract

The transport of nutrients by migratory animals across ecosystem boundaries can significantly enrich recipient food webs, thereby shaping the ecosystems’ structure and function. To illustrate the potential role of islands in enabling the transfer of matter across ecosystem boundaries to be gauged, we investigated the influence of seabirds on nitrogen input on islands. Basing our study on four widely differing islands in terms of their biogeography and ecological characteristics, sampled at different spatial and temporal intervals, we analyzed the nitrogen isotopic values of the main terrestrial ecosystem compartments (vascular plants, arthropods, lizards and rodents) and their relationship to seabird values. For each island, the isotopic values of the ecosystem were driven by those of seabirds, which ultimately corresponded to changes in their marine prey. First, terrestrial compartments sampled within seabird colonies were the most enriched in δ^15^N compared with those collected at various distances outside colonies. Second, isotopic values of the whole terrestrial ecosystems changed over time, reflecting the values of seabirds and their prey, showing a fast turnover throughout the ecosystems. Our results demonstrate that seabird-derived nutrients not only spread across the terrestrial ecosystems and trophic webs, but also modulate their isotopic values locally and temporally on these islands. The wealth of experimental possibilities in insular ecosystems justifies greater use of these model systems to further our understanding of the modalities of trans-boundary nutrient transfers.

## Introduction

Inputs of energy and nutrients in an ecosystem can exert a major influence on the dynamics of local populations, as well as the structure and evolution of recipient communities and food webs [1]–[5]. The particular mobility, behavior and physiology of biotic vectors can have a striking effect on the rate and type of exchanges across habitat boundaries [4], [6]. The ecological importance of nutrient transfers generated by species crossing boundaries between two ecosystems, whether for foraging or breeding, has long been recognized. Some clear examples of nutrient flows have been observed between aquatic and terrestrial ecosystems mediated by the trophic relationship between salmon and bears, *i.e*. [7], or between marine ecosystems and coastal ecosystems through inputs by nesting sea turtles [8], [9], by sea lions and whales [6], [10], or by terrestrial mammal predators of marine intertidal communities [11]. The flux of matter and energy across two ecosystems is especially important for small and closed ecosystems, such as islands. Given the size of their colonies, their wide distribution and the large amount of marine biomass that seabirds deposit on islands *via* guano, feathers, carcasses or regurgitated marine prey [5], [12], [13], [14], breeding colonies of seabirds have been shown to have a major impact on terrestrial insular ecosystems.

Nitrogen stable isotope ratios serve as useful tools to trace marine inputs in terrestrial trophic webs, making it possible to trace bottom-up effects of nutrient input [15]. Seabird guano is enriched in ^15^N relative to ^14^N, partly due to the birds’ high position in the trophic chain [16] and to preferential volatilization of ^14^N from guano [17]. There is solid scientific literature demonstrating nitrogen enrichment of primary producers due to guano deposition across vegetal taxonomic groups (mosses, plants, algae; [18]–[21]). As a consequence, guano fertilization increases the primary productivity of plants, indirectly benefitting populations that consume detritus, plant tissues and seeds. This, in turn, facilitates high densities of their consumers’ predators [22]. In a more direct way, large marine-bird breeding colonies also increase the numbers of scavengers and predators, which feed directly on their carcasses [2], [22]. Thus, either directly or indirectly, seabirds alter the dynamics of terrestrial ecosystem compartments throughout the entire trophic web [23].

A comparison of the nutrient enrichment of islands with and without seabirds theoretically provides a means to demonstrate the contribution of seabirds to insular ecosystems. In this regard, the introduction of natural seabird predators can be seen as a large-scale ‘experiment’ that allows the study of the effects of seabirds on insular ecosystems. They can provide a means of comparing islands which have varying levels of seabird presence. In pioneer studies, Stapp et al. [24] and Stapp and Polis [3] demonstrated a seabird-derived ^15^N enrichment in native rodents and several arthropod groups (detritivores, herbivores and predators), while Markwell and Daugherty [22] found a ^15^N enrichment in lizards on seabird islands. More recently, predators introduced on New Zealand islands have been shown to disrupt below-ground and above-ground food webs *via* the suppression of the seabird-driven nutrient flow [25]–[27]. These innovative and pivotal studies have opened up a new avenue of research in insular ecosystem functioning. Understanding the extent and modalities of terrestrial trophic web enrichment by seabirds is essential to the proper understanding and conservation of insular biodiversity. If we are to gain a better understanding of these processes, we now need to generalize these studies by repeating them in contrasting ecological contexts, as well as characterize the processes by ascribing them to influences of ecosystem particularities.

As part of this new avenue of research, we performed two different but connected lines of research, illustrating them with a set of studies on the modulation of insular isotopic trophic webs by seabird-derived nutrients. We propose that such studies be conducted across different spatial and temporal scales at both the inter- and intra-island level, focusing on islands with different ecological characteristics (different latitudes, longitudes, geological origins and sizes). Because the seabird influence is likely to vary in space, different sampling sites with different bird densities on the same island would appropriately complement the biogeographic approach, thereby contributing to assess the spatial scale at which seabird nitrogen enrichment is effective. Lastly, temporal variations in the influence of seabirds on trophic webs would add to the panel of possible seabird-driven fluctuations in nutrient flows, thus allowing us to understand the timing, duration and amplitude of these effects.

**Figure 1:**
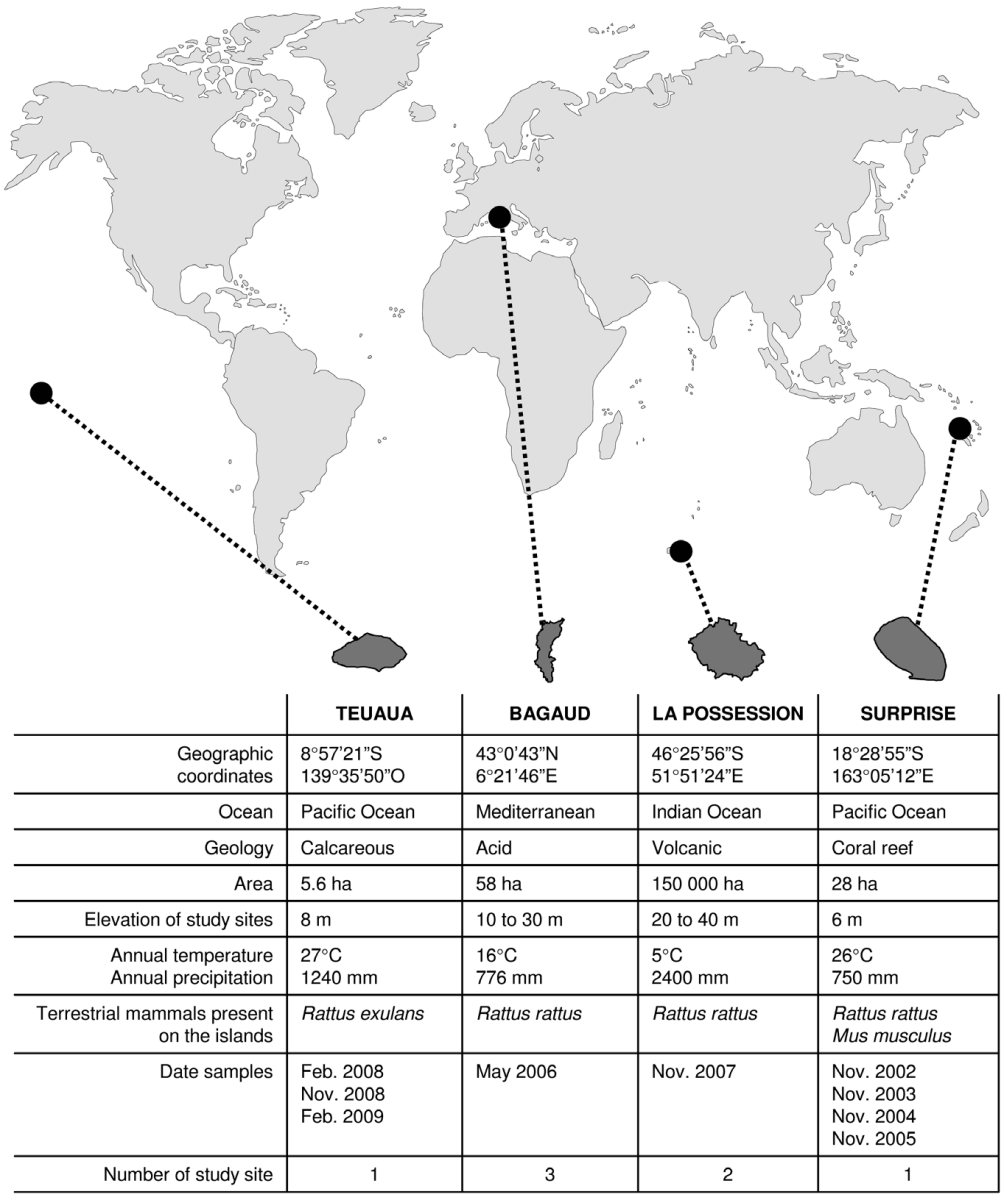
Characteristics of the four studied islands.

We illustrate this proposition with results from large-scale studies of the trophic webs of four islands with different biogeographic and ecological characteristics (Fig. 1), where changes were tracked over time (between 1 and 4 years) or, when possible, at different sites on each island,. Seabird nutrient input was tracked by measuring nitrogen isotopic values in seabirds and relating them to those of different ecosystem compartments, from plants to mammalian predators. We compared nitrogen isotopic values of species in each terrestrial ecosystem compartment (plants, arthropods and rodents): (1) between sites with different levels of seabird influence within islands (absence, presence, or sporadic presence of nesting seabirds) on Bagaud and La Possesion islands; (2) over time (to time the incorporation of seabird-derived nutrients into the trophic web) on Teuaua and Surprise islands. Although seabird nutrient input is expected to play a key role in the terrestrial ecosystem [24]–[27], there is a lack of isotopic studies exploring these two scales. We expected that spatial and temporal variations of δ^15^N values along the trophic webs (plants, arthropods, reptiles, rodents) of our various terrestrial ecosystems would be generated by the variations in the isotopic nitrogen values of seabirds on these islands.

## Materials and Methods

### Study Sites

Sampling was carried out on four islands located in four different oceans and seas, and differing widely in latitude, longitude, area and geology: Teuaua, Surprise, La Possession and Bagaud (Fig. 1). Surprise and La Possession are isolated oceanic islands, Bagaud is coastal, and Teuaua lies close to Ua Huka, a larger, inhabited island of the Marquesas Archipelago. As all are currently uninhabited, human activity may be supposed to have few effects on the nitrogen enrichment, and all four islands have an ecosystem that consists of at least 3 different terrestrial compartments, including plants, arthropods and rodents (*Rattus* spp. and sometimes *Mus musculus*).

Animals in this study were humanely treated according to the French and Overseas French Territories legislations (Décret n°2003-768/NOR: AGRD0300394D). Stephane Caut was authorized by the French Minister of Agriculture (R-45GRETA-F1-04). Different institutional review boards approved the sampling for each island: on La Possesion Island sampling was approved by the Scientific Committee of the French Polar Institute (Pr. No. 136); on Teuaua islet, sampling was performed within the context of a Pacific rat eradication attempt (“Restoration of Important Pacific Seabird Islands - Phase 1" program) approved by the local municipality of Ua Huka (Grant 2006–30661 and 30662) and Direction Régional de l’Environnement (DIREN) of the Gouvernement Territorial de la Polynésie Française; on Bagaud island, which is a strict Nature Reserve managed by the Part-Cros National Park (French ministry for the environment), licenses and permission to work, handle and collect were issued and approved by the prefecture of Var (authorisation No. 7/2004) and by the authorities of the Port-Cros National Park (programmes 08.031.83400 and 10-006 83400 PC); and sampling on Surprise Island was approved by the Gouvernement de la Nouvelle-Calédonie (CS05-7000-1044).

### Sampling the Ecosystem Compartments

All islands were sampled using the same protocol and most of the authors worked on at least two islands in order to minimize sampling variability.

In order to study the spatial effect of seabirds at a smaller spatial scale (*i.e*., within an island), the two larger islands, Bagaud and La Possession, were sampled at three and two sites, respectively, characterized by different levels of seabird influence (absent, present or sporadic). As Teuaua and Surprise are small islands characterized by a high density of breeding seabirds which occupied the total area of the islands during the breeding period, we focused on illustrating temporal variations.

Possession Island was sampled in November-December 2007 at the beginning of the seabird breeding period. Bagaud Island was sampled during May 2006 in the middle of the seabird breeding season. From 2002 to 2005, Surprise Island was sampled yearly, in early November, when most breeding bird species are present. Teuaua Islet was sampled in February 2008 when the islet was totally bird-free immediately after the breeding season, and in November 2008 and in February 2009, when the islet hosted a very large breeding population of sooty terns, *Onychoprion fuscatus* [28]. Moreover, these seabird breeding periods corresponded to of the peak activity of the ecosystems (e.g. plant growth, lizard activity and rodent reproduction), generally before the dry season.

Two sites were selected in the north-eastern part of La Possession Island, according to the presence or absence of seabirds [29]: (i) American Bay, which hosts a permanent rookery of king penguins, *Aptenodytes patagonicus* (density 0.8 to 1.2 breeding pairs/m^2^, [29]), and three above-ground ecosystem compartments besides that of the seabirds: vascular plants, arthropods (Amphipoda, Arachnida, Coleoptera, Diptera, Lepidoptera), and the black rat, (*Rattus rattus*); (ii) a second site located 4 km north-west of the former site and 1.5 km away from the sea coast (Hébé Bay), totally devoid of bird colonies and hosting three ecosystem compartments: plants (mosses and vascular plants), arthropods (Arachnida, Coleoptera, Diptera, Lepidoptera), and the black rat.

Three sites were sampled in the southern part of Bagaud Island, according to different levels of seabird influence: the Gull site, which hosts an important breeding colony of yellow-legged gulls *Larus michahellis* (density 1.0 breeding pairs/m^2^), the Scrubland site (200 m away) without seabirds, and a coastal site located 150 m away from the Gull site and 50 m away from the Scrubland site, where seabirds nested or rested sporadically (density 0.7 breeding pairs/m^2^) [30]. Besides the seabird compartment, the same ecosystem compartments were identified at the three sites: plants (vascular plants), arthropods (Isopoda, Arachnida, Coleoptera, Dermaptera, Hemiptera, Hymenoptera, Lepidoptera, Orthoptera) and the black rat.

We sampled the centre of the small Surprise Island, where various species of seabirds breed: the red-footed booby, *Sula sula*, the brown booby, *S. leucogaster*, the masked booby, *S. dactylatra*, the brown noddy, *Anous stolidous*, and the black noddy, *A. minutus* (yearly recorded breeding pairs/100 m^2^ between 2002 and 2005: 1.1, 0.6, 1.1, 0.8). We identified five different ecosystem compartments in addition to that of the seabirds: plants, arthropods, (Orthoptera, Coleoptera and Lepidoptera), reptiles (strand litter skink *Caledoniscincus haplorhinus* and mourning gecko *Lepidodactylus lugubris*), and rodents (black rat and house mouse, *Mus musculus*).

Lastly, Teuaua Islet hosts one of the largest breeding sooty tern colonies (1.2 to 1.8 breeding pairs/m^2^ [28]) in French Polynesia, a species which does not exhibit strict seasonality in reproduction. Besides the seabird compartment, we identified four other ecosystem compartments: plants, arthropods (Coleoptera and Blattaria), reptiles (mottled snake-eyed skink, *Cryptoblepharus poecilopleurus*), and rodent (Polynesian rat, *Rattus exulans*).

On each island we took into account most of the above-ground ecosystem compartments, including fauna and flora. Tissue samples of all plant species were collected on each island and at each study site. Ground arthropods were captured with pitfall traps or by hand and the whole body was used for isotopic analysis. Reptiles were caught by hand and a tail muscle sample was collected. Seabird samples consisted of muscle tissue from freshly-dead seabirds or abandoned eggs found in the colony. Rats and mice were captured with live traps or snap traps, depending on the island. The seabird prey provided by seabird regurgitates (65% Exocoetidae, 26% Cephalopoda and 9% indeterminate fishes; n = 23) were collected on Surprise and Teuaua islands. All samples were stored in 70% ethanol. Some species identifications were performed or confirmed in the laboratory before stable isotope analysis (see [31] for further information).

### Isotope Analyses

All samples were dried at 60°C for 48 h, ground to a fine powder, weighed in tin capsules and stored in a desiccator until isotope measurement. Isotope analyses were performed using an IsoPrime spectrometer (*MicroMass*, Service Central d’Analyse, Solaize, France) coupled to a EuroEA 3024 analyzer. Stable N isotope ratios are expressed as:
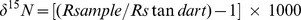
Where *R* is ^15^N/^14^N. The standard for the N isotopic ratio is IAEA-N1 (+0.4‰) and IAEA-N2 (+20.3‰). Replicate assays of internal laboratory standards indicated measurement maximum errors (SD) of ±0.2‰.

### Data Analyses

To test the effect of seabird presence on the nitrogen isotopic values of the different ecosystem compartments of La Possession and Bagaud islands, we performed one ANOVA for each island. δ^15^N values were treated as the dependent variable, while the site (two and three sites on La Possession and Bagaud islands, respectively) was treated as the independent variable. On Bagaud Island, a post-hoc contrast analysis was performed to identify significant differences among the three sites [32]. Although only one analysis was performed for each island, separate analyses for each ecosystem compartment were carried out (specifying a “by" option [33]) within each island.

To test the effects of seabird isotopic values on the values of the different ecosystem compartments of Surprise and Teuaua islands, we performed one ANOVA for each island. We tested whether δ^15^N values differed among ecosystem compartments, dates and the interaction between both factors (trends in δ^15^N values vary in the same (or opposite) direction). A non-significant interaction would mean that the isotopic signatures of all ecosystem compartments, including seabirds and seabird prey, follow the same trend over time.

Data were analyzed with General Linear Models (GLM, SAS v.8.2, PROC GENMOD, with “dscale" option as the scaled deviance differed from unity). Normality of variables was tested and all models were fitted specifying a normal distribution with identity link function [33].

## Results

### Global Patterns

**Figure 2:**
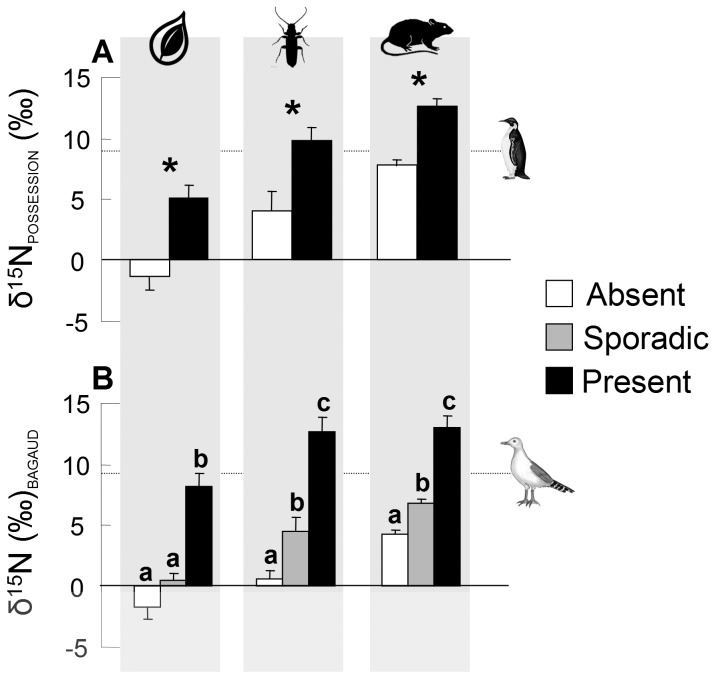
Mean (+SE) δ^15^N values of the ecosystem compartments for different levels of seabird influence; on (A) La Possession Island and (B) Bagaud Island. Within each island, each ecosystem compartment is represented by a symbol at the top and was analyzed separately: plants, arthropods and rodents. In (A) asterisks represent significant differences between bars within each compartment. Bars sharing a common letter were not significantly different in (B) based on contrast analyses. The dotted line with the seabird symbol (penguin for La Possession Island and seagull for Bagaud Island) represents the mean isotopic value of seabirds on each island.

**Figure 3:**
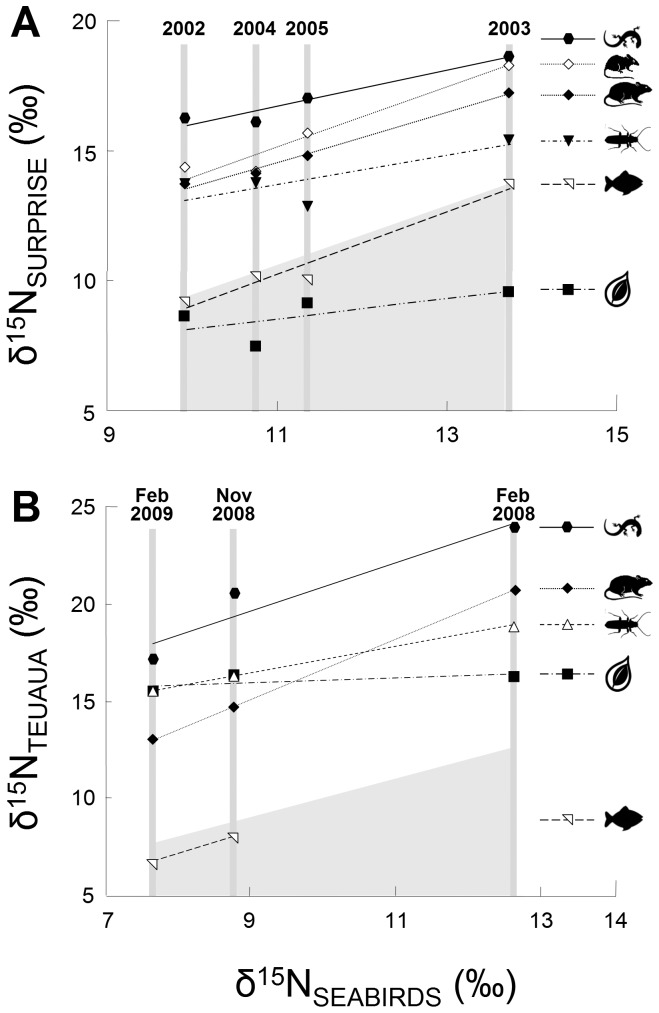
Relationships between seabird δ^15^N values and δ^15^N values of each ecosystem compartment; on (A) Surprise Island and (B) Teuaua Island. Symbols represent each ecosystem compartment: plants, seabird prey, arthropods, rats, mice and reptiles. Seabird values are shown by the upper edge of the grey polygon.

Analyses clearly show that for all islands (at all sites and on all sampling dates, Fig. 1, Table S1), the nitrogen isotopic values of the ecosystem depended on those of seabirds, which ultimately corresponded to changes in their marine prey. In fact, the transfer of nitrogen along trophic webs (Fig. 2, 3) was driven by birds. These results are similar and show the same trend for the different ecosystems studied. This congruence of results across islands of widely diverging biogeography is strengthened by the results on spatial and temporal variations of the influence of seabirds in insular communities.

### Spatial Influence of Seabirds on δ^15^N Values

Sampling at different sites of La Possession and Bagaud islands revealed that δ^15^N values of all terrestrial ecosystem compartments were significantly higher at sites where seabirds were breeding than at sites where they were absent or only sporadically present (Fig. 2, Table S1). On La Possession Island, significantly higher δ^15^N values were consistently found between the seabird colony site and the non-breeding site for the three terrestrial ecosystem compartments (F_1,25_ = 18.28, p<0.001, *n* = 31 for plants; F_1,13_ = 9.39, p = 0.002, *n* = 15 for arthropods; F_1,25_ = 26.99, p<0.001, *n* = 27 for rodents; Fig. 2A), despite the close proximity (*i.e.*, 4 km) of the two sites. Similarly, on Bagaud Island, where the three study sites (at which seabirds were present, absent or sporadically present) were even shorter distances apart, *i.e*, 50–200 m, significant differences in the δ^15^N values of the three terrestrial ecosystem compartments were found among sites (F_2,39_ = 28.16, p<0.001, *n* = 42 for plants; F_2,25_ = 36.74, p<0.001, *n* = 28 for arthropods; F_2,42_ = 71.84, p<0.001, *n* = 45 for rodents; Fig. 2B, Table S1). Post-hoc contrast analysis revealed a spatial gradient in δ^15^N values: the highest δ^15^N were found at the seabird breeding site, whereas the lowest δ^15^N were found where seabirds were absent. The third site, where seabirds only nested and rested sporadically, had intermediate δ^15^N values (Fig. 2B). These differences were significant for all terrestrial ecosystem compartments except plants, where values between the two sites that were less influenced by seabirds did not significantly differ (Fig. 2B).

### Temporal Influence of Seabirds on δ^15^N Values

Sampling during the seabird breeding season in different years on Surprise Island revealed differences in δ^15^N values among ecosystem compartments (including seabirds and their marine prey) and also among years (2003–2005), but the interaction between both factors was not significant (F_6,228_ = 145.35, p<0.001 for compartments; F_3,228_ = 32.74, p<0.001 for dates; and F_18,228_ = 1.55, p = 0.063, for the interaction, *n* = 256; Fig. 3B). Mean δ^15^N values were the lowest in 2002 and highest in 2003, but were intermediate in 2004 and 2005 (Table S1). The fact that both factors (compartments and dates), but not their interaction, are significant, means that the trend over time was similar for the different ecosystem compartments.

On Teuaua Islet, we found the same trends in isotopic values among dates (δ^15^N values decreased from February 2008 to November 2009, Table S1) and among ecosystem compartments, but the interaction between compartments and dates was significant (F_4,50_ = 58.66, p<0.001; F_2,50_ = 47.04, p<0.001; and F_8,50_ = 5.65, p<0.001 for compartments, dates and their interaction, respectively, *n* = 65). The significant interaction was driven by the lowest compartments (plants and arthropods), which maintained more stable values over time (Fig. 3B). While rats and reptiles can incorporate seabird-derived nitrogen isotopes directly *via* predation, browsing or scavenging, plants and herbivorous arthropods incorporate them only indirectly through soil, which accounts for the smaller effect. Thus, the slopes of the relationships between the δ^15^N values of seabirds and those of their marine prey, reptiles and rats were similar, while the slopes of the relationship between the δ^15^N values of seabirds and those of plants and arthropods were smaller but still positive (Fig. 3B). In fact, if plants and arthropods were removed, the interaction would cease to be significant (F_2,39_ = 123.01, p<0.001 for compartments; F_2,39_ = 60.16, p<0.001 for dates; and F_4,239_ = 1.54, p = 0.210 for the interaction, *n* = 48).

Seabird values were always close to those of their marine prey (Fig. 3A, B). Mean seabird δ^15^N values were related to those of each ecosystem compartment, confirming that the main external nitrogen input in terrestrial compartments is due to seabirds and that δ^15^N values of all ecosystem compartments were driven by seabird δ^15^N values: plant compartments had the lowest mean δ^15^N values in the terrestrial ecosystem and tended to follow mean seabird values, especially on Surprise Island; variations in mean δ^15^N arthropod values followed mean seabird values more closely than plant values; and below seabird values were rat, mouse and reptile values, which closely followed seabird values, showing the same trend as seabirds (Fig. 3A, B).

It is interesting to note that on Teuaua Islet the nitrogen isotopic values of all ecosystems decreased over time (from Feb. 2008 to Feb. 2009), while on Surprise Island values first increased (from 2002 to 2003), then decreased (between 2003 and 2004) and finally increased again (from 2004 to 2005) (see order of survey dates at the top of Fig. 3A, B). From these results, it is clear that the influence of seabirds on δ^15^N values varies with time, causing a greater or lesser general nitrogen enrichment of the ecosystem, depending on each case, but either increases or decreases the whole ecosystem’s δ^15^N values.

## Discussion

Using a multitrophic level perspective, we highlighted the potential of using insular ecosystems with varying ecological characteristics to assess the modalities of nutrient transfer by seabirds into terrestrial ecosystems. Illustrating our study with multiple site or date sampling on four contrasting islands, we showed that the input of nutrients by seabirds rapidly and globally modulates the isotopic values of terrestrial ecosystems. First, our results confirm that nitrogen enrichment by marine inputs enriches primary and secondary consumers (*i.e*., plants, arthropods, reptiles, mice and rats). Second, we showed that the extent of marine nutrient spill-over into inland areas could be restricted to very small spatial scales. This could have major implications for field sampling. Third, we highlighted that temporal changes in seabird isotopic nitrogen values could induce rapid change in the rest of the isotopic terrestrial ecosystem. The patterns described are reinforced by the similarity of our results in geographically distant islands with different trophic structure, climate conditions and oceanic influences.

### Temporal Variation of Marine and Seabird δ^15^N Values

The effects of seabirds as vectors of marine-derived nutrients to terrestrial ecosystems have been reported previously, especially focusing on the effects on soil and plants (see *e.g*., [25], [34]–[37], but they have also been studied at higher trophic levels such as invertebrates [22], [24], [38], lizards [22], [39] and rodents [3], [25]–[27], [30] (see [40] for review). On the whole, however, few studies have assessed the temporal variation of nitrogen isotopic values derived from seabird nutrients (e.g. [3], [5], [24], [30]). Seabirds always enrich the recipient ecosystems with nitrogen, but nitrogen enrichment is not always necessarily associated with nitrogen isotopic enrichment. Indeed, we show here for the first time that nitrogen isotopic changes could be in either direction; that is, isotopic values of the ecosystem could be enriched, impoverished or both, over time, following the isotopic values of seabirds and their prey. There were significant variations in the isotopic values of seabirds (and their prey) from year to year. It is well known that ocean primary production varies significantly over time [41]. Such variations are translated to the marine food web and subsequently enter the terrestrial food webs *via* the prey of seabirds [14].

Moreover, values changed within a few months, between the arrival of seabirds and the sampling time, when all compartments were active before the dry season. The time it takes for the isotopic values of a prey to be incorporated into the tissues of the consumer - i.e., the isotopic turnover – usually ranges from a week to several months, depending on the species (for the tissues with a high turnover rate, [15]). Similarly, from a multitrophic point of view, we showed a δ^15^N turnover of the ecosystem, that is, the time it took for biovector-derived nutrients to be incorporated across the entire trophic web (e.g. [42]). The ecosystem turnover found in our study was unexpectedly rapid (e.g. a few months on Teuaua). It should be noted that climate very likely influences the speed and pathways of ^15^N’s transfer from guano to plants and/or soil. The ecosystem turnover would, therefore, appear to depend not only on trophic web length and complexity, but also on other biogeographic factors, such as climate and substrate.

### The Importance of the Spatial Scale

It should be taken into account that the mobility of biotic vectors also affects the spatial influence of nutrient transport [6]. Indeed, the effects of nutrient input from seabirds may operate on much larger spatial scales than that from animals with low within-island mobility (*e.g.*, marine turtles [8] and penguins [43]). Because ammonia can be volatilized into the atmosphere from seabird colonies and deposited at sites far removed from colonies, guano deposition has been shown to have far-reaching effects [43], [44]. In contrast, our results showed the markedly localized effect of isotopic enrichment by birds on Bagaud Island, where highly significant differences in isotopic nitrogen values were recorded in areas only 50–200 m apart (*e.g*., plants ∼8‰, Fig. 2). Most previous studies considered the effects of seabirds at relatively small spatial scales (*i.e.*, within a colony or in an adjacent area) or generalized enrichment patterns at the island scale, comparing islands with and without seabirds (*e.g*. [25]). However, a few studies suggest that examining larger scales within islands may reveal very different patterns [21]. Our results demonstrate the importance of the spatial scale, as well as the temporal scale, especially regarding sampling selection, when defining the impact of seabirds on the trophic web through stable isotopes.

### Ecological Implications

The fact that seabirds modulate multiple ecosystem compartments indirectly shows that seabird-derived nutrients play a major role in the structure, dynamics and abundance of species. For example, as guano increases plant and animal biomass and changes species composition [18]–[21], [45], [46], it might affect high trophic levels (*e.g.*, the composition and dynamics of animal communities). In their review, Kolb et al. [40] revealed how the bottom-up effects of seabirds propagate up trophic chains to increase populations of a variety of island consumers under different environmental conditions. Thus, any fluctuation in the populations of seabirds, or even in their marine prey, can lead to important chain reactions in the overall functioning of the terrestrial ecosystem. Much recent progress has been made regarding the potential consequences of the widespread disappearance of seabirds, especially as a result of the introduction of alien predators [13], [47], [48]. Fukami et al. [25], Wardle et al. [26] and then Mulder et al. [27] showed how the presence or absence of seabirds, due to alien invasive rodents, can alter entire communities and even favor the establishment of new alien plants. In this context, there is an urgent need to further investigate how current declines in seabird populations might affect nutrient deposition or even have unanticipated top-down or bottom-up consequences as a result of trophic cascades. Conversely, large-scale demographic explosions, as in some seagull species [49], can trigger substantial changes in the composition of island biotic communities, for example by favoring the establishment of invasive plants [49]–[53] or improving the survival of invasive rodents. Knowledge on isotopic variations over time and space due to seabird nutrient input will contribute to the interpretation of the seabirds’ potential role in ecosystem communities.

In the present study, using nitrogen isotope tracers, we confirmed that the input of seabird-derived marine nutrients is central to the dynamics of nitrogen isotopic values of the above-ground trophic web. We have also shown that this influence is detectable and variable at very small spatial and temporal scales. Seabirds are only a biovector transferring nutrients from marine to terrestrial ecosystems, so that changes in the latter depend on changes in the former [24]. Further studies are needed to understand the modalities of such isotopic transfers according to biogeography and community characteristics. In this context, islands can serve as potent tools towards a better understanding of this key process.

## Supporting Information

Table S1 **Nitrogen isotopic values (δ^15^N(‰), mean±SE) of each ecosystem compartment for the four different islands, and sites and sampling dates within islands**. For Arthropods, each sample represented a pool of different individuals of the same species.
(DOC)
