# Supplementary material for: Seabird Modulations of Isotopic Nitrogen on Islands
Source: PLoS One. 2012 Jun 18;7(6):e39125. doi: 10.1371/journal.pone.0039125 (PMC3377609; doi:10.1371/journal.pone.0039125)
Supplement: Table S1 — Nitrogen isotopic values (δ15N(‰), mean±SE) of each ecosystem compartment for the four different islands, and sites and sampling dates within islands. For Arthropods, each sample represented a pool of different individuals of the same species. (DOC) [file pone.0039125.s001.doc]

**Table S1.** **Nitrogen isotopic values (δ^15^N(‰), mean ±SE) of each ecosystem compartment for the four different islands, and sites and sampling dates within islands**. For Arthropods, each sample represented a pool of different individuals of the same species.

l

**POSSESSION**

**Present**

**Absent**

|  |  |  |  |  |  |  |  |  |  |  |  |
| --- | --- | --- | --- | --- | --- | --- | --- | --- | --- | --- | --- |
|  |  |  |  |  |  |  |  |  |  |  |  |
| Compartment | Tissu |  |  | *n* | δ^15^N | SD |  |  | *n* | δ^15^N | SD |
|  |  |  |  |  |  |  |  |  |  |  |  |
|  |  |  |  |  |  |  |  |  |  |  |  |
| *Rattus rattus* | Liver |  |  | *12* | 12.39 | 0.77 |  |  | *15* | 7.85 | 0.49 |
| Plants | Leaf |  |  | *18* | 5.16 | 1.05 |  |  | *13* | -1.42 | 1.08 |
| Arthropods | Whole body |  |  | *8* | 9.95 | 1.11 |  |  | *7* | 4.07 | 1.62 |
| Seabirds | Muscle |  |  | *3* | 8.79 | 0.10 |  |  | *-* |  |  |
|  |  |  |  |  |  |  |  |  |  |  |  |

**BAGAUD**

**Present**

**Sporadic**

**Absent**

|  |  |  |  |  |  |  |  |  |  |  |  |  |  |  |  |  |
| --- | --- | --- | --- | --- | --- | --- | --- | --- | --- | --- | --- | --- | --- | --- | --- | --- |
|  |  |  |  |  |  |  |  |  |  |  |  |  |  |  |  |  |
| Compartment | Tissu |  |  | *n* | δ^15^N | SD |  |  | *n* | δ^15^N | SD |  |  | *n* | δ^15^N | SD |
|  |  |  |  |  |  |  |  |  |  |  |  |  |  |  |  |  |
|  |  |  |  |  |  |  |  |  |  |  |  |  |  |  |  |  |
| *Rattus rattus* | Liver |  |  | *7* | 12.88 | 1.07 |  |  | *18* | 6.78 | 0.31 |  |  | *20* | 4.18 | 0.39 |
| Plants | Leaf |  |  | *14* | 8.14 | 1.13 |  |  | *6* | 0.35 | 0.62 |  |  | *22* | -1.78 | 0.87 |
| Arthropods | Whole body |  |  | *12* | 12.56 | 1.31 |  |  | *4* | 4.39 | 1.16 |  |  | *11* | 0.50 | 0.71 |
| Seabirds | Muscle |  |  | *4* | 9.34 | 0.45 |  |  | *-* |  |  |  |  | *-* |  |  |
|  |  |  |  |  |  |  |  |  |  |  |  |  |  |  |  |  |


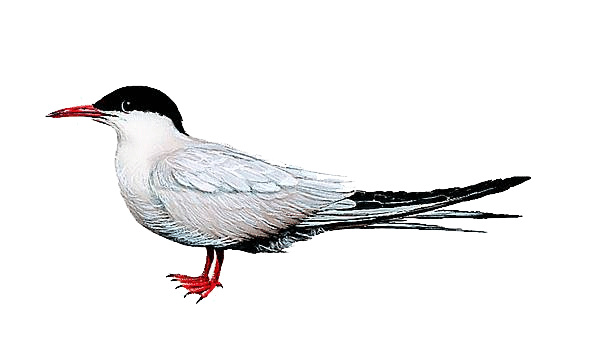


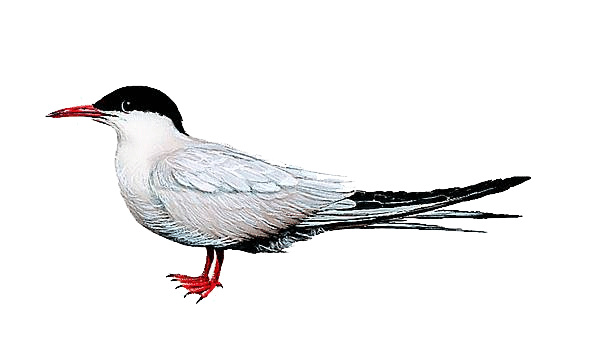


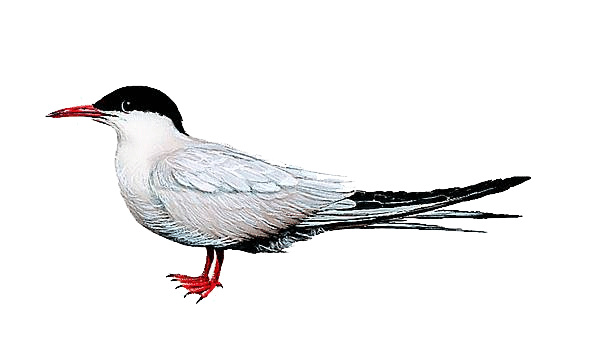


**TEUAUA**

**Feb-2008**

**Nov-2008**

**Feb-2009**

|  |  |  |  |  |  |  |  |  |  |  |  |  |  |  |  |  |
| --- | --- | --- | --- | --- | --- | --- | --- | --- | --- | --- | --- | --- | --- | --- | --- | --- |
|  |  |  |  |  |  |  |  |  |  |  |  |  |  |  |  |  |
| Compartment | Tissu |  |  | *n* | δ^15^N | SD |  |  | *n* | δ^15^N | SD |  |  | *n* | δ^15^N | SD |
|  |  |  |  |  |  |  |  |  |  |  |  |  |  |  |  |  |
|  |  |  |  |  |  |  |  |  |  |  |  |  |  |  |  |  |
| *Rattus exulans* | Muscle |  |  | *18* | 20.58 | 0.40 |  |  | *1* | 14.62 | - |  |  | *16* | 12.97 | 0.14 |
| Plants | Leaf |  |  | *2* | 16.12 | 1.01 |  |  | *2* | 16.37 | 0.49 |  |  | *4* | 15.37 | 0.45 |
| Arthropods | Whole body |  |  | *3* | 18.82 | 1.51 |  |  | *3* | 16.35 | 0.41 |  |  | *3* | 15.59 | 0.86 |
| Reptiles | Muscle |  |  | *2* | 23.77 | 0.81 |  |  | *2* | 20.39 | 0.19 |  |  | *2* | 17.03 | 0.50 |
| Seabird prey | Muscle |  |  | *-* |  |  |  |  | *3* | 7.99 | 0.58 |  |  | *2* | 6.64 | 0.60 |
| Seabirds | Muscle |  |  | *2* | 12.65 | 0.88 |  |  | *3* | 8.79 | 0.45 |  |  | *2* | 7.67 | 0.11 |
|  |  |  |  |  |  |  |  |  |  |  |  |  |  |  |  |  |


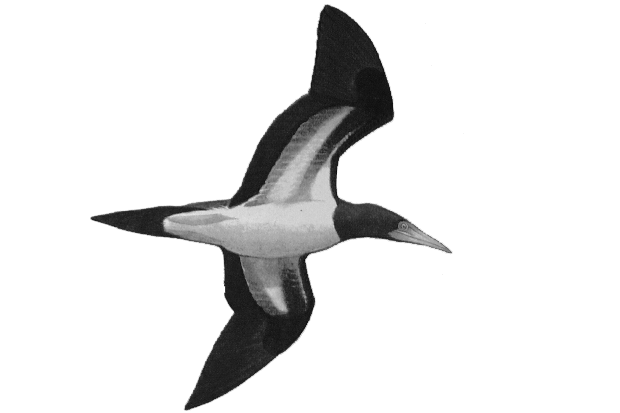


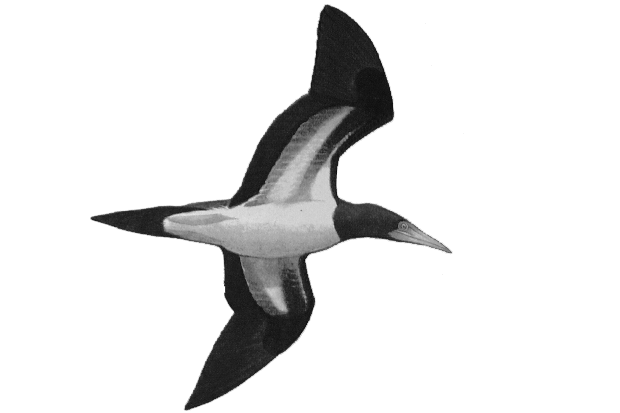


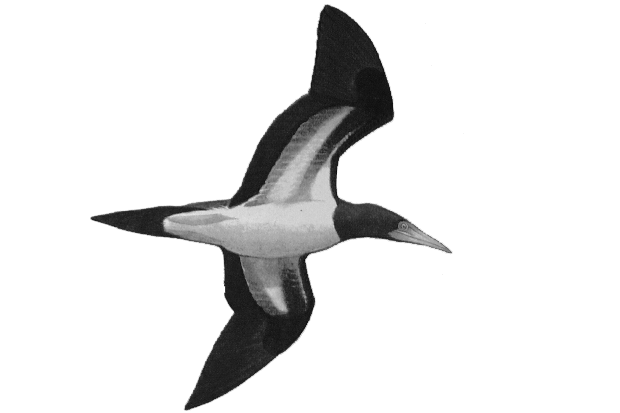


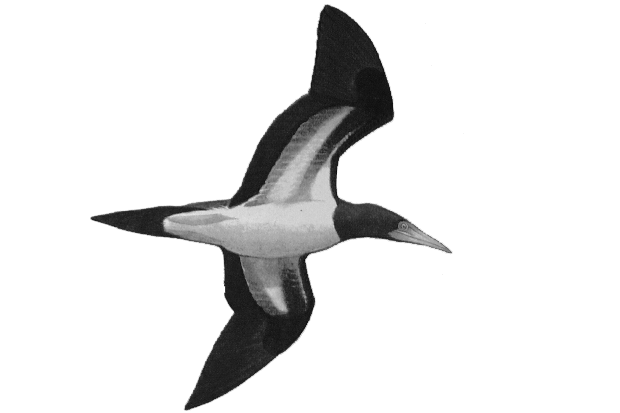


**SURPRISE**

**Nov-2002**

**Nov-2003**

**Nov-2004**

**Nov-2005**

|  |  |  |  |  |  |  |  |  |  |  |  |  |  |  |  |  |  |  |  |  |  |
| --- | --- | --- | --- | --- | --- | --- | --- | --- | --- | --- | --- | --- | --- | --- | --- | --- | --- | --- | --- | --- | --- |
|  |  |  |  |  |  |  |  |  |  |  |  |  |  |  |  |  |  |  |  |  |  |
| Compartment | Tissu |  |  | *n* | δ^15^N | SD |  |  | *n* | δ^15^N | SD |  |  | *n* | δ^15^N | SD |  |  | *n* | δ^15^N | SD |
|  |  |  |  |  |  |  |  |  |  |  |  |  |  |  |  |  |  |  |  |  |  |
|  |  |  |  |  |  |  |  |  |  |  |  |  |  |  |  |  |  |  |  |  |  |
| *Rattus rattus* | Liver |  |  | *5* | 13.71 | 0.39 |  |  | *3* | 17.27 | 1.29 |  |  | *5* | 14.09 | 0.33 |  |  | *10* | 14.78 | 0.24 |
| *Mus musculus* | Liver |  |  | *2* | 14.46 | 0.22 |  |  | *8* | 18.46 | 0.38 |  |  | *10* | 14.97 | 0.27 |  |  | *7* | 15.67 | 0.47 |
| Plants | Leaf |  |  | *21* | 8.65 | 0.57 |  |  | *23* | 9.58 | 0.40 |  |  | *22* | 7.47 | 0.53 |  |  | *22* | 9.14 | 0.34 |
| Arthropods | Whole body |  |  | *12* | 13.79 | 0.56 |  |  | *9* | 15.44 | 0.46 |  |  | *8* | 13.76 | 0.88 |  |  | *7* | 12.87 | 0.74 |
| Reptiles | Muscle |  |  | *8* | 16.23 | 0.10 |  |  | *9* | 18.42 | 0.42 |  |  | *7* | 16.15 | 0.42 |  |  | *10* | 17.07 | 0.63 |
| Seabird prey | Muscle |  |  | *8* | 9.15 | 0.46 |  |  | *3* | 13.73 | 0.54 |  |  | *4* | 10.03 | 0.54 |  |  | *3* | 9.93 | 0.58 |
| Seabirds | Muscle |  |  | *12* | 9.93 | 0.28 |  |  | *19* | 13.73 | 0.24 |  |  | *6* | 10.77 | 0.33 |  |  | *5* | 11.39 | 0.10 |
|  |  |  |  |  |  |  |  |  |  |  |  |  |  |  |  |  |  |  |  |  |  |
